# Clinical Significance of sIL-2R Levels in B-Cell Lymphomas

**DOI:** 10.1371/journal.pone.0078730

**Published:** 2013-11-13

**Authors:** Noriaki Yoshida, Miyo Oda, Yoshiaki Kuroda, Yuta Katayama, Yoshiko Okikawa, Taro Masunari, Megumu Fujiwara, Takashi Nishisaka, Naomi Sasaki, Yoshito Sadahira, Keichiro Mihara, Hideki Asaoku, Hirotaka Matsui, Masao Seto, Akiro Kimura, Koji Arihiro, Akira Sakai

**Affiliations:** 1 Department of Hematology and Oncology, Research Institute Radiation Biology and Medicine, Hiroshima University, Hiroshima, Japan; 2 Division of Molecular Medicine, Aichi Cancer Center Research Institute, Nagoya, Japan; 3 Department of Cancer Genetics, Nagoya University Graduate School of Medicine at Aichi Cancer Center Research Institute, Nagoya, Japan; 4 Department of Anatomical Pathology, Hiroshima University, Hiroshima, Japan; 5 Department of Internal Medicine, Hiroshima Red Cross Hospital & Atomic-Bomb Survivors Hospital, Hiroshima, Japan; 6 Department of Hematology, National Hospital Organization Kure Medical Center, Kure, Japan; 7 Department of Hematology, Chugoku Central Hospital, Fukuyama, Japan; 8 Department of Pathology, Hiroshima Red Cross Hospital & Atomic-Bomb Survivors Hospital, Hiroshima, Japan; 9 Department of Laboratory Pathology, Hiroshima Prefecture Hospital, Hiroshima, Japan; 10 Department of Pathology, Kure Kyosai Hospital, Kure, Japan; 11 Department of Pathology, Kawasaki Medical School, Kurashiki, Japan; 12 Department of Molecular Oncology & Leukemia Program Project, Research Institute for Radiation Biology and Medicine, Hiroshima University, Hiroshima, Japan; 13 Department of Laboratory medicine, Kure Kyosai Hospital, Kure, Japan; 14 Department of Radiation Life Science, Fukushima Medical University, Fukushima, Japan; The University of North Carolina at Chapel Hill, United States of America

## Abstract

Elevated soluble interleukin-2 receptor (sIL-2R) in sera is observed in patients with malignant lymphoma (ML). Therefore, sIL-2R is commonly used as a diagnostic and prognostic marker for ML, but the mechanisms responsible for the increase in sIL-2R levels in patients with B-cell lymphomas have not yet been elucidated. We first hypothesized that lymphoma cells expressing IL-2R and some proteinases such as matrix metalloproteinases (MMPs) in the tumor microenvironment can give rise to increased sIL-2R in sera. However, flow cytometric studies revealed that few lymphoma cells expressed IL-2R α chain (CD25) in diffuse large B-cell lymphoma (DLBCL) and follicular lymphoma (FL), and most CD25-expressing cells in the tumor were T-cells. Distinct correlations between CD25 expression on B-lymphoma cells and sIL-2R levels were not observed. We then confirmed that MMP-9 plays an important role in producing sIL-2R in functional studies. Immunohistochemical (IHC) analysis also revealed that MMP-9 is mainly derived from tumor-associated macrophages (TAMs). We therefore evaluated the number of CD68 and CD163 positive macrophages in the tumor microenvironment using IHC analysis. A positive correlation between the levels of sIL-2R in sera and the numbers of CD68 positive macrophages in the tumor microenvironment was confirmed in FL and extranodal DLBCL. These results may be useful in understanding the pathophysiology of B-cell lymphomas.

## Introduction

Serum soluble interleukin-2 (IL-2) receptor (sIL-2R) was discovered in supernatants of adult T-cell leukemia/lymphoma (ATLL) cell lines [1], and now has been recognized as a tumor-related biomarker of malignant lymphomas, including B-cell malignancies [2], [3]. IL-2 receptor comprises three different IL-2 receptor chains: α, β, and γ. Among these, the α (CD25) on the cell membrane is cleaved by proteolytic processing, and the cleaved α chain is detected as sIL-2R [4]. The ligand of IL-2R, IL-2, plays a critical role in the development of T and NK lymphocyte as a growth factor.

ATLL is a peripheral T-cell neoplasm caused by human T-cell leukemia virus type 1 (HTLV-1). Tumor cells are characterized by CD4 and CD25 positivity on their cell membranes. Therefore, sIL-2R is thought to reflect tumor burden because of the expression of CD25 [3], [5]. Recently, sIL-2R has been shown to have predictive value for patients with acute type and lymphoma type ATLL [6].

Diffuse large B-cell lymphoma (DLBCL) and follicular lymphoma (FL) are the first and second common lymphomas in B-cell lineage. There is a consensus that international prognostic index (IPI) and follicular lymphoma international prognostic index 2 (FLIPI2) are prognostic factors in DLBCL and FL, respectively [7]–[11]. In B-cell malignancies, sIL-2R was first recognized in patients with hairy cell leukemia in which leukemia cells are positive for CD25 [12]. Thereafter, elevated sIL-2R was also detected in the sera of patients with DLBCL and FL, and several reports confirmed that sIL-2R levels are related to the prognosis in lymphoma [13]–[15]. However, the mechanisms of sIL-2R elevation in patients with B cell lymphomas and ATLL remain to be clarified.

Several studies have indicated that matrix-metalloproteinase 9 (MMP-9), a member of the MMP family, has the ability to cleave IL-2R α chain [16], [17]. MMPs are important proteolytic enzymes involved in cancer metastasis and invasion owing to disruption of extracellular matrix (ECM) [18]–[20]. Some reports have showed that ATLL cells produce MMP-9, and that expression levels of MMP-9 are related to organ involvement and tumor progression [21], [22]. Previous studies of gene expression profile have also revealed that tumor-associated macrophages (TAM) primarily express MMP-9 in DLBCL [23].

In this study, we first analyzed whether sIL-2R is a significant prognostic factor in DLBCL and FL. Subsequently, we hypothesized that lymphoma cells expressed IL-2R and proteinases such as matrix metalloproteinases (MMPs) in the tumor microenvironment could give rise to increased sIL-2R in sera. Based on these hypotheses, we analyzed whether MMP-9 cleaves IL-2R α chains and which cell types produce MMP-9 in tumors, and we analyzed the relationships between levels of sIL-2R in sera and the number of tumor-associated macrophages. The results suggest that the number of CD68-positive macrophages that produce MMP-9 is associated with high levels of sIL-2R.

## Materials and Methods

### Ethics Statement

The samples and the medical records used in our study were approved by the Institutional Review Board (IRB) at Hiroshima University and Chugoku Central Hospital. Our study was limited to the use of excess human tissue samples and clinical courses; therefore, the IRB exempted the need for written consent from the patients. Written informed consent was obtained from all participants for further analysis of lymph nodes samples.

### Relationship between concentration of sIL-2R and prognosis in DLBCL and FL

One hundred and four patients with DLBCL and thirty patients with FL were diagnosed between November 2000 and December 2007 at Hiroshima University Hospital and Chugoku Central Hospital. All patients received chemotherapy and Rituximab (R)+CHOP (cyclophosphamide, doxorubicin, vincristine and prednisolone) therapy or R+THP-COP (pirarubicin, cyclophosphamide, vincristine, and prednisolone) therapy. We determined overall survival (OS) for these patients. OS was calculated from the date of diagnosis to death or the last date of follow-up. Differences in OS according to sIL-2R levels were evaluated by Wilcoxon test. A p-value <0.05 was considered to be statistically significant.

### Phenotype analysis

Biopsy samples of lymph nodes were minced and evaluated by two-color flow cytometry. Antibodies used in this study were: (1) FITC-CD19 (Becton Dickinson, Fukushima, Japan) and PE-CD25 (BD Biosciences, San Jose, CA); (2) FITC-CD3 (Becton Dickinson) and PE-CD25 (BD Biosciences). Immunofluorescence of the labeled cell membrane was evaluated using a FACS Calibur flow cytometer (Becton Dickinson, Franklin Lakes, NJ).

### Measurement of sIL-2R in sera of ML patients and supernatants of cell lines

Sera from untreated patients were used for measurement of sIL-2R. Cells (5×10^6^/ml) of CD25-positive ATLL cell line (MT4) were cultured in fetal cow serum (FCS)-free RPMI 1640, which were then treated with 400 ng/ml recombinant human MMP-9 (rMMP-9) (R & D Systems,Inc., Minneapolis, MN) or MMP-9 inhibitor (Calbiochem, San Diego, CA). After 6 h of culture, supernatants were measured for sIL-2R using chemiluminescent enzyme Immunoassay (CLEIA) (Siemens Healthcare Diagnostics, Tokyo, Japan). Results were determined in triplicate and Wilcoxon signed-rank test was used to analyze the differences.

### Expression of CD25 after treatment with recombinant MMP-9

A total of 1×10^6^/ml MT4 cells, an ATLL cell line were treated with rMMP-9 for 6 h and subsequently analyzed for CD25 by flow cytometry. Experiments were performed in triplicate.

### Measurement of MMP-9 in patient plasma

Plasma samples from patients with malignant lymphoma or lymphoproliferative disease were used for measurement of MMP-9 by ELISA (GE Healthcare, Piscataway, NJ), after informed consent.

### Immunohistochemistry to detect MMP-9, CD68, and CD163 expression

Samples for histological diagnosis were formalin-fixed, paraffin-embedded and stained using the hematoxylin-eosin method. Paraffin sections from each sample were immunostained with mouse monoclonal antibodies against MMP-9 (Thermo Fisher Scientific, San Diego, CA), CD163 (Clone 10D6) (Thermo Fisher Scientific), and CD68 (KP-1) (DakoCytomation, Glostrup, Denmark) (see details in File S1, Methods section).

### Counting of TAMs and relationship between number of TAMs and levels of sIL-2R in DLBCL, FL and RLH

In order to calculate the number of macrophages in the tumor microenvironment, we performed IHC studies of 53 DLBCL, 20 FL and 12 lymph node hyperplasia (RLH), using CD68 and CD163 monoclonal antibodies. Counting of these macrophages was conducted in accordance with a previous report [24]. Briefly, macrophage counts were estimated 5 times in high-power fields (×400 magnification). Mean counts were then determined and used for statistical analysis. In DLBCL, we counted whole macrophages in samples. In FL and RLH controls, we counted intrafollicular macrophages, as a previous result showed that the number of intrafollicular CD68-positive macrophages reflects prognosis of patients with FL [24]. Only areas containing tumors were analyzed for the IHC study, and areas containing only necrosis and fibrosis were excluded. Neutrophils were also excluded. Differences in the number of macrophages in each disease and the relationship between levels of sIL-2R and number of macrophages were evaluated by Mann-Whitney U test and Spearman's rank correlation coefficient, respectively.

## Results

### sIL-2R as prognostic factor in patients with DLBCL and FL

**Figure 1:**
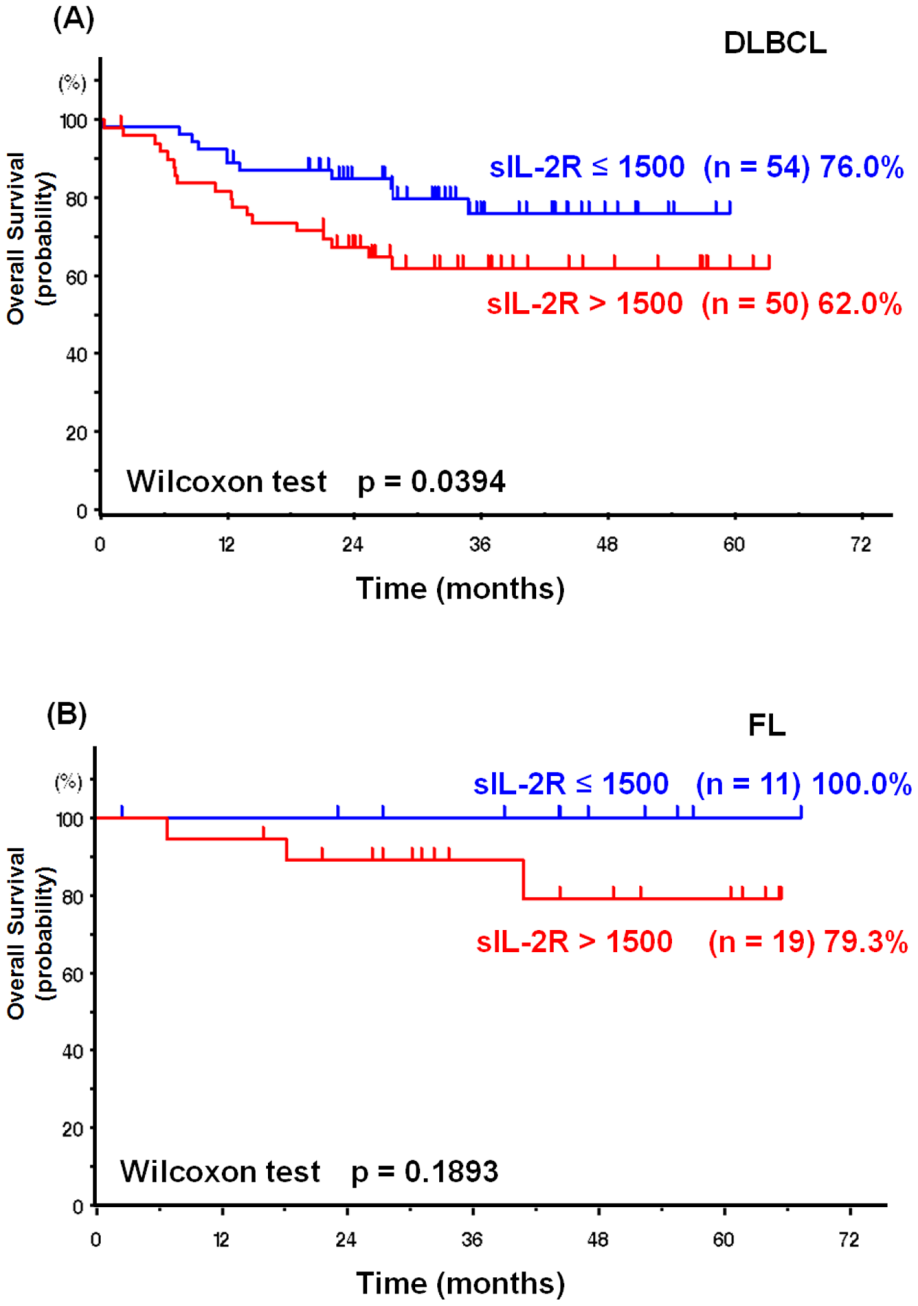
Overall survival based on sIL-2R levels (≤1500 vs. >1500) in DLBCL (A) and FL (B). Serum sIL-2R levels were analyzed in previously untreated patients with DLBCL (n = 104) or FL (n = 30). The 5-year OS rates for patients with sIL-2R levels of ≤1500 U/ml and >1500 U/ml were 76% and 62%, respectively (p<0.05) in DLBCL, and 100% and 79.3%, respectively (p = 0.189) in FL.

In order to determine whether sIL-2R has prognostic value in B-cell lymphomas, we retrospectively analyzed 104 DLBCL and 54 FL cases. Patients were divided into two groups according to pre-treatment sIL-2R concentration (>1500 and ≤1500) [25]. In DLBCL, patients with high sIL-2R had poor prognosis when compared to patients with low sIL-2R (p<0.05) (Figure 1A). Patients with high sIL-2R in FL tended to have poor prognosis, although the difference did not reach significance (p = 0.1893) (Figure 1B). Furthermore, no FL patients with low sIL-2R died. Thus, sIL-2R is a useful prognostic factor in DLBCL as well as in FL.

### Diversity of CD25 expression in lymphoma cells

We aimed to determine why elevated sIL-2R was observed in patients with B-cell lymphomas, particularly in patients with poor prognosis. If the levels of sIL-2R reflect tumor burden, expression of CD25 in CD19-positive lymphoma cells may be related to sIL2-R. Thus, we performed a flow cytometric study on a fraction of samples of DLBCL, FL, mantle cell lymphoma (MCL) and RLH, using the two-color staining method with antibodies against CD19/CD25 and CD3/CD25.

**Figure 2:**
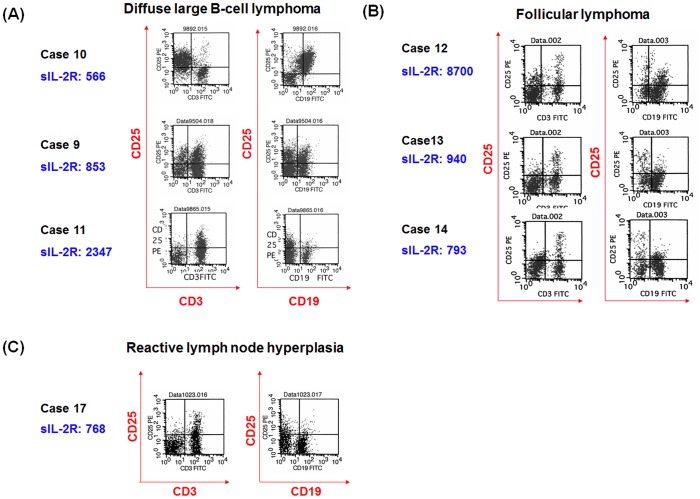
Representative expression of CD25 (IL-2Rα) on lymphoma cells and normal T-cells in diffuse large B cell lymphoma, follicular lymphoma, and mantle cell lymphoma. Patient numbers are based on Table 1. Representative cases in diffuse large B cell lymphoma (DLBCL) (A), follicular lymphoma (FL) (B) and reactive lymph node hyperplasia (RLH) (C). There was no apparent association between CD25 expression on lymphoma cells (CD19-positive) or T-cells (CD3-positive) and levels of sIL-2R in these cases.

Representative flow cytometry data are shown in Figures 2A–2C. In DLBCL, most lymphoma cells (CD19-positive cells) were positive for CD25 and T-cells (CD3-positive cells) were positive for CD25 partially in Case 10, while T-cells and lymphoma cells were partially positive for CD25 in Case 9, and T-cells were partially positive for CD25 and most lymphoma cells were negative for CD25 in Case 11. In FL, T-cells were partially positive for CD25 in all cases and lymphoma cells were partially positive in Case 12 or mostly negative for CD25 in Cases 13 and 14. In reactive lymph node hyperplasia (RLH), T-cells were partially positive for CD25 and B-cells (CD19-positive cells) were mostly negative for CD25. On the other hand, two of the two MCL tumor cells were positive for CD25 (Table 1 and Figure S1 in File S1).

**Table 1:** Relationship between sIL-2R concentrations and expressions of CD25.

No	Diagnosis	sIL-2R (U/ml)	LDH (IU/l)	CS	IPI*	CD25 on B cell	CD25 on T cell
1	DLBCL	4350	250	IVB	H	(−)	(+)
2		5180	574	IIIB	H-I	(−)	(+)
3		760	156	IA	L	(−)	(+)
4		3520	339	IVB	H	(+)	scattered
5		3420	199	IVA	H-I	(−)	scattered
6		6960	295	IIIA	H-I	(+)	(+)
7		56000	497	IIIB	H-I	(+)	(+)
8		653	197	IVB	H-I	(+)	(+)
9		853	197	IIIA	H	(+)	(+)
10		566	195	IVA	L-I	(+)	scattered
11		2347	282	IVA	H	(−)	(+)
12	FL	8700	194	IVB	I	(+)	(+)
13		940	135	IVA	I	(−)	(+)
14		793	149	IIIA	H	(−)	(+)
15	MCL	1380	151	IA	L	(+)	(+)
16		5130	257	IIIA	H-I	(+)	(+)
17	RLH	768	NA			(−)	(+)

* FLIPI1 was used in patients with FL.

Abbreviations: sIL-2R; soluble interleukin-2 receptors, LDH; lactate dehydrogenase, CS; clinical stage, IPI; international prognostic index, DLBCL; diffuse large B-cell lymphoma, FL; follicular lymphoma, MCL; mantle cell lymphoma, RLH; reactive lymph node hyperplasia, NA; not available.

Normal range: sIL-2R; 145–518 U/ml, LDH; 119–229 IU/l.

Next, we analyzed the relationship between levels of sIL-2R in sera and CD25 expression on lymphoma cells or on T-cells, and other clinical data (Table 1). Particularly in DLBCL, levels of sIL-2R were less than 1000 U/ml even in patients with advanced clinical stage and CD25-positive lymphoma cells (Cases 8, 9 and 10), while levels of sIL-2R were more than 3000 U/ml in patients with advanced clinical stage, but not CD25-positive lymphoma cells (Cases 1, 2 and 5). Therefore, we speculated that sIL-2R levels in serum do not precisely indicate tumor burden as a majority of tumor cells did not express CD25 in DLBCL.

### Matrix metalloproteinase-9 (MMP-9) cleavage of IL-2Rα chains

Expression of CD25 in tumor cells did not clarify the mechanisms responsible for elevation of sIL-2R in B-cell lymphomas. Thus, we hypothesized that some proteinases produced by tumor cells or bystanders cleave IL-2Rα chains. Interestingly, MMP-9 is reported to have the ability to cleave IL-2Rα chains [16], [17].

**Figure 3:**
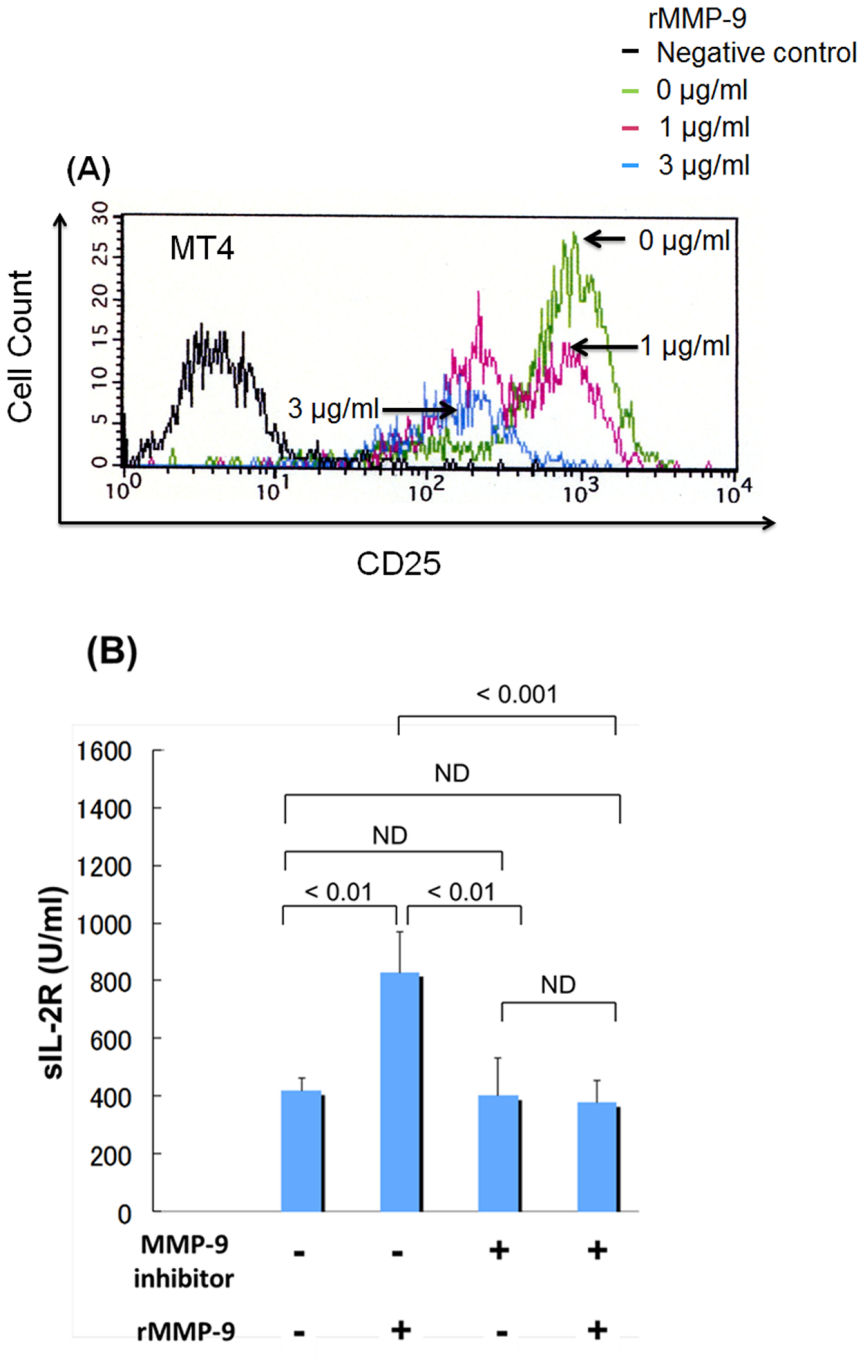
Effects of MMP-9 on cleavage of IL-2Rα. (A) The ATL cell line, MT4, in which MMP-9 expression was not detected by RT-PCR, was treated with rMMP-9 (1 µg/ml or 3 µg/ml). After 6 h of incubation, analysis of CD25 expression was performed by flow cytometry. (B) MT4 was treated with rMMP-9 (400 ng/ml) or MMP-9 inhibitor (0.1 mM), and cultured in FCS-free medium. After 6 h of culture, experiments were performed in triplicate. P-values of <0.05 were considered to be statistically significant.

First, we investigated whether IL-2Rα chains on lymphoma cells are cleaved by MMP-9. We were unable to obtain B-cell lymphoma cell lines that were positive for CD25; therefore an ATL cell line (MT4) was used in this analysis [26]. MT4 cells did not express endogenous MMP-9, and other CD25-positive cell lines express endogenous MMP-9 (Figure S2 in File S1). We analyzed expression of CD25 by flow cytometry after 6 h of incubation of MT4 with recombinant MMP-9 (1 µg/ml, 3 µg/ml) in FCS-free medium. Treatment with 1 µg/ml rMMP-9 partially decreased expression of CD25, and treatment with 3 µg/ml rMMP-9 markedly decreased expression of CD25 in almost all cells (Figure 3A). Therefore, MMP-9 is able to cleave IL-2Rα chains depending on its concentration. Subsequently, MT4 cells were treated with 400 ng/ml rMMP-9 with or without MMP-9 inhibitor in FCS-free medium. After 6 h of treatment, we then measured sIL-2R in the supernatants of MT4 cells. Levels of sIL-2R in supernatants increased with rMMP-9 treatment, but decreased with MMP-9 inhibitor treatment as compared to those of sIL-2R treated with rMMP-9 groups (Figure 3B). However, sIL-2R was also detected in the supernatants of control groups, which did not express endogenous MMP-9. This raised the possibility that factors in addition to MMP-9 are involved in the cleavage of sIL-2R. Collectively, we confirmed that MMP-9 plays an important role in the cleavage of IL-2Rα chains.

### Levels of sIL-2R and MMP-9 in serum

As it has been demonstrated that MMP-9 cleaves IL-2Rα chains, we then analyzed whether levels of sIL-2R are correlated with those of MMP-9 in patients with DLBCL and FL. We analyzed patient plasma for measurement of MMP-9 as neutrophils and platelets produce their own MMP-9 during the procedure for serum collection [27], [28].

**Figure 4:**
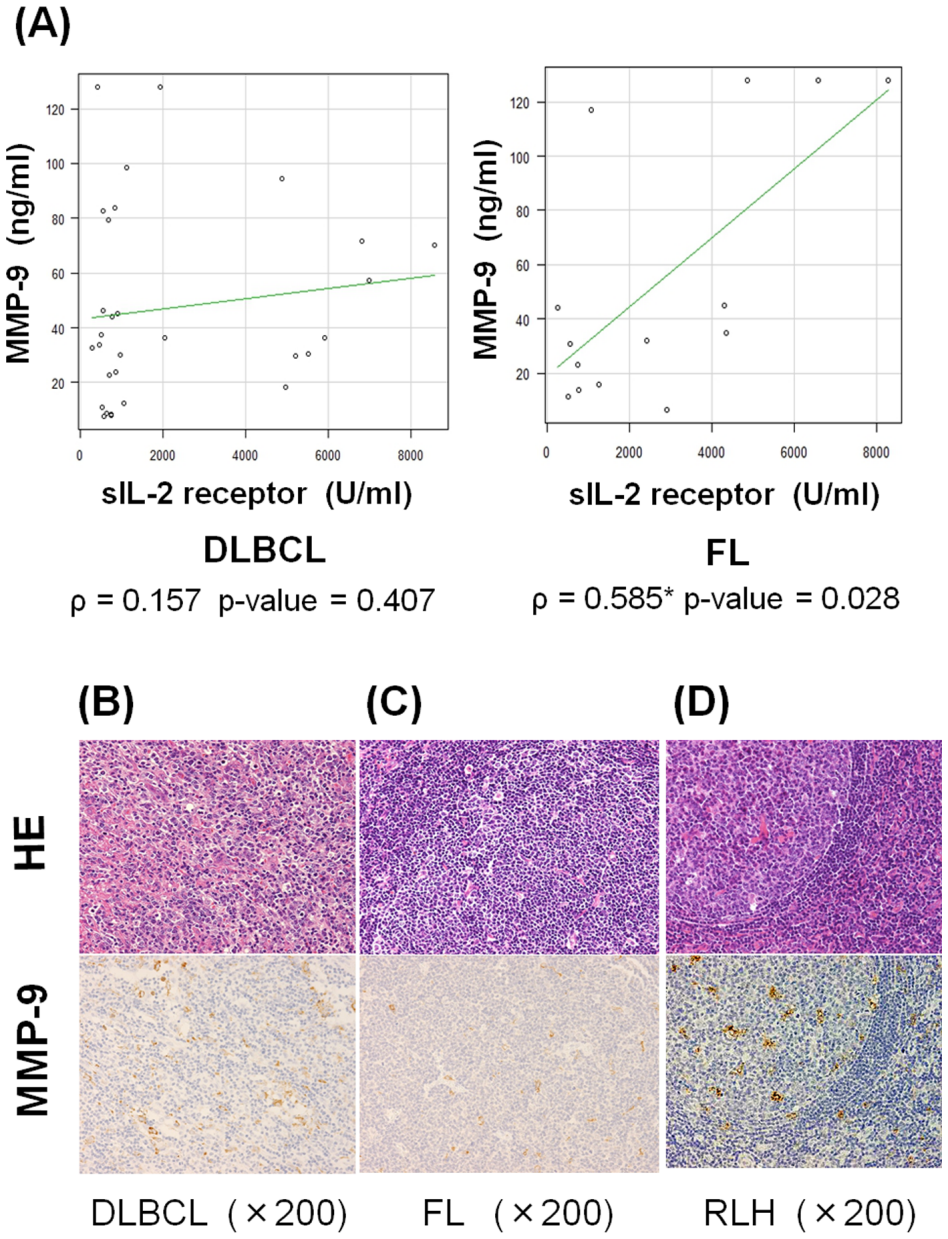
Production of MMP-9 by tumor-associated macrophages. (A) Positive correlations between sIL-2R and MMP-9 levels were observed in patients with FL (ρ = 0.585, p-value = 0.028), but not in DLBCL. (B) Immunohistochemical staining with anti-MMP-9 antibody in DLBCL, FL and RLH. MMP-9-positive cells were mainly macrophages in each sample, and lymphoma cells were negative for MMP-9 in DLBCL and FL. *Significant correlation was observed.

We analyzed levels of sIL-2R and MMP-9 in untreated patients (30 DLBCL and 14 FL) (Table S1 in File S1). The average levels of sIL-2R were 2228 (range, 304–8560) U/ml and 2775 (range 268–8237) U/ml in DLBCL and FL, respectively, and the average concentrations of MMP-9 were 47.2 (7.68–128) ng/ml and 34.1 (6.4–128) ng/ml in DLBCL and FL, respectively. Correlations between levels of sIL-2R and MMP-9 were evaluated using Spearman's rank correlation coefficient. In FL, there was a positive correlation (ρ = 0.585, p-value = 0.028), but not in DLBCL (ρ = 0.157, p-value = 0.407) (Figure 4A). Next, we purified B-cells from lymph nodes of DLBCL, FL and RLH using CD19 microbeads. Cells were cultured in FCS-free RPMI-1640, and we then analyzed the activity of MMP-9 in the supernatants by gelatin zymography. We detected MMP-9 activity in the B-cells of each lymph node (Figure S3 in File S1). Thus, we confirmed that B-cells express MMP-9 and then attempted to confirm the expression of MMP-9 by means of IHC. However, tumor-associated macrophages (TAMs), but not lymphoma cells, were positive for MMP-9 (Figures 4B–D). Furthermore, all B-cell lymphoma cells, including intravascular B-cell lymphoma (IVL), were negative for MMP-9, while ATLL cells were positive for MMP-9 (Figure S4 in File S1). These results indicate that macrophages, but not lymphoma cells, mainly express MMP-9, which cleaves IL-2Rα chains in lymph node lesions.

### Positive correlations between levels of sIL-2R and number of CD68-positive macrophages

We assumed that the MMP-9 produced by intratumoral macrophages plays a crucial role in elevation of sIL-2R; thus, we calculated the number of MMP-9-positive cells in an IHC study. However, no correlations between numbers of MMP-9-positive cells and levels of sIL-2R were observed (data not shown). Therefore, we intensively analyzed the number of tumor-associated macrophages (TAM), because they produce MMP-9 and are reportedly correlated with poor prognosis in some solid tumors and lymphoma [23], [24], [29]–[31].

**Figure 5:**
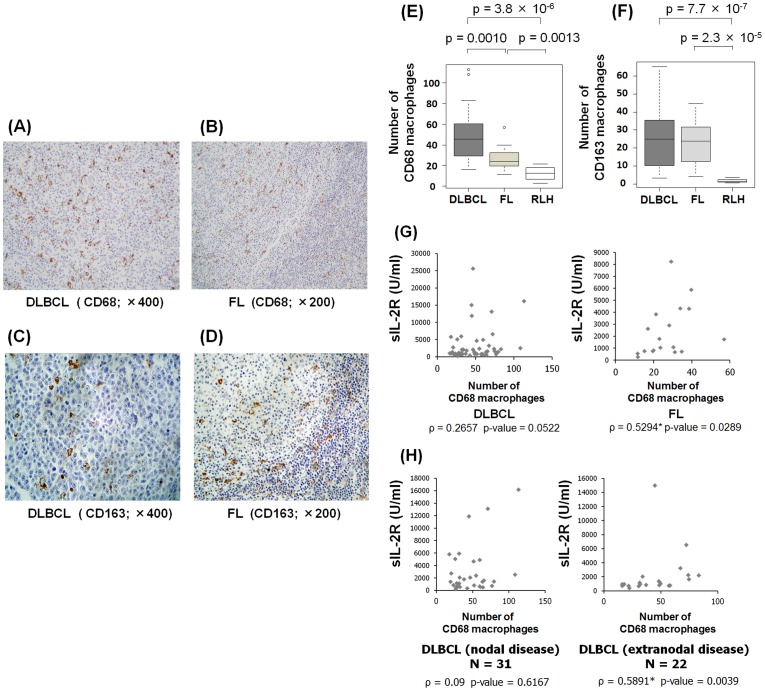
Positive correlation between number of CD68-positive macrophages and sIL-2R levels. We counted the number of CD68- or CD163-positive macrophages in DLBCL, FL and RLH. Representative images of CD68-positive or CD163-positive macrophages in DLBCL and FL are shown (A–D). We also analyzed the correlations between number of CD68-positive macrophages and sIL-2R levels in DLBCL, FL and RLH. Intrafollicular macrophages were counted in FL and RLH. In both DLBCL and FL, the number of CD 68-positive macrophages was higher than that in RLH. (E) In both DLBCL and FL, the number of CD163-positive macrophages was significantly higher than that in RLH. (F) There was a positive correlation between the number of CD68-positive macrophages and sIL-2R levels in FL (right, ρ = 0.5294, p-value = 0.0289) (G). There was also a positive correlation between the number of CD68-positive macrophages and sIL-2R concentrations in extranodal samples of DLBCL. (right, ρ = 0.5891, p-value = 0.0039) (H). *Significant correlations were observed.

First, we counted the number of TAMs using CD68 and CD163 antibodies (Figures 5A–D); subsequently, we analyzed the correlations between the number of TAMs and levels of sIL-2R. CD68 is a marker of pan-macrophages, and previous reports demonstrated that increases of intrafollicular CD68-positive macrophages are related to poor prognosis in FL and classical Hodgkin's lymphoma (CHL) [24], [31]. CD163 is also a marker of macrophages, and previous reports revealed that the number of CD163-positive macrophages reflects prognosis in angioimmunoblastic T-cell lymphoma (AITL) [29], [30].

Patient information and numbers of macrophages are shown in Table 2. The number of CD68-positive macrophages in DLBCL and FL was significantly higher than that of RLH (Figure 5E). Similarly, the number of CD163-positive macrophages in DLBCL and FL was significantly higher than that of RLH (Figure 5F). Based on these results, we analyzed the correlations between the number of CD68-positive or CD163-positive macrophages and the levels of sIL-2R in DLBCL and FL. Interestingly, there was a positive correlation between the number of CD68-positive macrophages and the levels of sIL-2R in FL (ρ = 0.5284, p-value = 0.0289), but not in DLBCL (ρ = 0.2657, p-value = 0.0522) (Figure 5G). The number of CD163-positive macrophages was not correlated with the levels of sIL-2R in DLBCL and FL (data not shown). As the DLBCL samples analyzed in this study comprised 31 cases of nodal disease, and 22 cases of extranodal disease (e.g., skin, intestine and soft tissue), we analyzed the numbers of CD68-positive and CD163-positive macrophages in lymph nodes and extra lymph nodes, separately. There was a positive correlation between levels of sIL-2R and number of CD68-positive macrophages in extranodal DLBCL (ρ = 0.5891, p-value = 0.0039), but not in nodal DLBCL (ρ = 0.09, p-value = 0.6167) (Figure 5H). The number of CD163-positive macrophages was not associated with levels of sIL-2R in either nodal or extranodal DLBCL (data not shown). However, the numbers of CD68-positive and CD163-positive macrophages were not associated with patient prognosis in DLBCL (including extranodal samples) or FL (Figure S5 in File S1).

**Table 2:** Characteristics of analyzed patients and results of immunohistochemical study.

Disease	site	No. of cases	Age, median (range)	sIL-2R, median (range)	No. of CD68-macrophages median (range)	No. of CD163-macrophages median (range)
DLBCL		54	69 (35–86)	1313.5 (217–25600)	45.7 (16–113.7)	25 (3.2–65.2)
	nodal	31	67 (35–85)	1404 (217–16150)	45 (17.8–113.7)	27.8 (3.2–65.2)
	extranodal	23	72 (44–86)	1013 (402–25600)	46.6 (16–83.2)	20.6 (3.2–61)
FL		19	55 (26–82)	1742 (268–71900)	23.8 (11.6–57)	23.8 (4.2–44.6)
RLH	intrafollicular	11	54 (22–71)	472.5 (185–3923)	12.8 (2.8–21.6)	1.6 (0.6–3.4)

Abbreviations: sIL-2R; Soluble Interleukin-2 Receptor, DLBCL; Diffuse large B-cell lymphoma, FL; Follicular lymphoma, RLH; reactive lymph node hyperplasia.

In summary, CD68-positive macrophages in the tumor microenvironment may be a factor in the elevation of sIL-2R in FL and may play a role in extranodal DLBCL.

## Discussion

### Ability of MMP-9 to cleave IL-2Rα chain

In contrast to ATLL, the majority of B-cell lymphomas do not express CD25 on the cell surface; however, sIL-2R is related to poor prognosis in B-cell malignancies, particularly in DLBCL. Based on the results of flow cytometry, the number of tumor cells or normal T lymphocytes expressing CD25 did not reflect sIL-2R levels. This indicates the existence of other factors responsible for production of sIL-2R, and we considered MMP-9 to be the main factor responsible for production of sIL-2R through cleavage of the IL-2R α chain expressed on lymphoma cells and bystander T-cells. To characterize the functional effects of MMP-9 on IL-2R cleavage, MT4 with CD25 on their surfaces and not showing endogenous MMP-9 were analyzed (Figure S2 in File S1). Experiments in such cells cultured with rMMP-9 and MMP-9 inhibitor confirmed MMP-9 cleavage of IL-2Rα chains on malignant lymphoma cells. Expression of MMP-9 in B-cells containing lymphoma cells in DLBCL and FL was detected on gelatin zymography, but not on IHC. On the other hand, IHC study revealed that the macrophages in the tumor microenvironment were slightly positive for MMP-9 in samples of DLBCL and FL samples. Therefore, the main source of MMP-9 could be macrophages and B-cells containing lymphoma cells are also the producer of MMP-9 in tumors.

### Contribution of CD68-positive macrophages to elevation of sIL-2R

Several reports have shown that TAMs are related to poor prognosis and tumor progression in malignant tumors, including lymphoma [24], [30]–[33], and we also believed that TAMs are responsible for production of sIL-2R as they were positive for MMP-9 on IHC analysis. Interestingly, we found that there were positive correlations between the number of CD68-positive macrophages and the levels of sIL-2R in FL and extranodal DLBCL. On the other hand, the numbers of CD163-positive macrophages are not related to the levels of sIL-2R. Previous data of gene expression profiling of macrophages and monocytes was adapted in an effort to determine whether the expression levels of CD68 are correlated with those of MMP-9 [34]. The results revealed positive correlations between the expression levels of CD68 and those of MMP-9 (Figures S6 in File S1), thus the number of CD68-positive macrophages may be correlated with sIL-2R levels. In addition, the expression levels of other enzymes (for example, MMP-2 and PLAU) were also correlated with those of CD68 in macrophages/monocytes (Figure S6A in File S1) [34]. Among them, MMP-2, which is a gelatinase containing gelatin binding repeat as well as MMP-9, is thought to have the effect of cleavage of α chain of IL-2R [35], although the expressions of MMP-2 were lower than those of MMP-9 (Figures S2 and S3 in File S1). Since these enzymes may affect sIL-2R levels, no apparent correlations between sIL-2R levels and MMP-9 levels may be observed in patients with DLBCL and FL (Table S1 in File S1).

### TAMs in DLBCL

DLBCL is a heterogeneous group of B-cell lymphoma, and is classified into activated B-cell (ABC)-type DLBCL, germinal center B-cell (GCB)-type DLBCL and primary mediastinal B-cell type DLBCL based on gene expression profile [36]. According to the Hans algorithm of DLBCL [37], our study includes 9 cases of GCB-type DLBCL and 2 cases of non-GCB type DLBCL. However, apparent correlations were not observed among the types of DLBCL, and the number of CD68-positive and CD163-positive macrophages, and sIL-2R levels (Figures S7A–B in File S1). Previous gene expression profiling data for DLBCL was used to analyze this correlation [38]. The results indicated that gene expression related to TAMs [34] was present in each type of DLBCL, and MYC expression levels were not associated with this gene expression (Figure S7C in File S1). We therefore speculate that TAMs are unaffected by the differentiation status of B-cell lymphoma cells, and do not affect the type of DLBCL.

### sIL-2R levels reflect the tumor microenvironment

**Figure 6:**
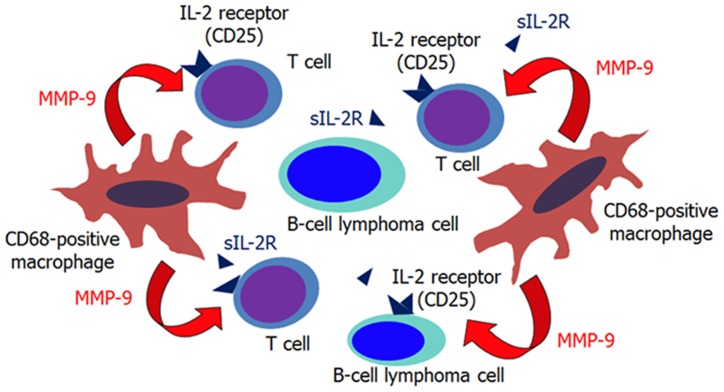
Model of sIL-2R elevation in B-cell lymphomas. MMP-9 released from tumor associated macrophages (especially CD68-positive) cleaves the IL-2R α chain on bystander T-cells and B-lymphoma cells, if they express CD25 (IL-2Rα). This mechanism may be involved in elevation of sIL-2R levels in patients with B-cell lymphomas.

In this study, we combined the results of flow cytometry, IHC, sIL-2R levels, and MMP-9 levels to examine the mechanisms responsible for variations in sIL-2R levels in lymphoma. We then speculate that MMP-9 is a crucial factor to produce sIL-2R and that the main source of MMP-9 is TAMs (Figure 6), although the number of analyzed cases was relatively small. It is also conjectured that sIL-2R levels reflect the status of tumor microenvironment components in lymphoma. Furthermore, our speculation leads to an explanation why sIL-2R level is high in patients with RLH and other chronic inflammatory disease: sIL-2R is released from activated T cells mainly due to the cleavage by proteinases containing MMP-9 produced by inflammation related cells.

However, the significance of sIL-2R in sera is complex because CD25 is mainly expressed on activated T-cells and is partially expressed on lymphoma cells in FL and DLBCL; furthermore, there exists CD25-positive DLBCL (Case 10 in Figure 2A) and lymphoma cell also produces MMP-9 (Figure S3 in File S1). Therefore, MMP-9 produced by B-cell lymphoma cells may also contribute to the increase of sIL-2R levels in sera. Therefore, sIL-2R/IL-2 interaction might be a significant factor with regard to tumor burden and tumor microenvironment. Although several questions remain, the present results indicate the involvement of TAMs in B-cell lymphomas. These findings would therefore be of great help in revealing the pathophysiology of B-cell lymphomas.

## Supporting Information

File S1 **File S1 includes the following:** Methods. Supplemental Methods are described in this section. Figure S1. Expression of CD25 (IL-2Rα) in MCL. Figure S2. MMP-9 expression in ATL cells. Figure S3. MMP-9 expression in CD19-positive cells from B-cell lymphoma and RLH. Figure S4. Immunohistochemical staining in intravascular large B cell lymphoma (IVL) and adult T-cell leukemia/lymphoma (ATLL) using anti-MMP-9 antibody. Figure S5. Overall survival based on numbers of CD68 and CD163 positive macrophages in DLBCL total, extranodal DLBCL and FL. Figure S6. Gene expression profiles of macrophages and monocytes. Figure S7. Tumor associated macrophages in diffuse large B-cell lymphoma. Table S1. sIL-2R and MMP-9 concentrations in DLBCL and FL.
(PDF)
