# Supplementary material for: Clinical Significance of sIL-2R Levels in B-Cell Lymphomas
Source: PLoS One. 2013 Nov 13;8(11):e78730. doi: 10.1371/journal.pone.0078730 (PMC3827264; doi:10.1371/journal.pone.0078730)
Supplement: File S1 — includes the following: Methods. Supplemental Methods are described in this section. Figure S1. Expression of CD25 (IL-2Rα) in MCL. Figure S2. MMP-9 expression in ATL cells. Figure S3. MMP-9 expression in CD19-positive cells from B-cell lymphoma and RLH. Figure S4. Immunohistochemical staining in intravascular large B cell lymphoma (IVL) and adult T-cell leukemia/lymphoma (ATLL) using anti-MMP-9 antibody. Figure S5. Overall survival based on numbers of CD68 and CD163 positive macrophages in DLBCL total, extranodal DLBCL and FL. Figure S6. Gene expression profiles of macrophages and monocytes. Figure S7. Tumor associated macrophages in diffuse large B-cell lymphoma. Table S1. sIL-2R and MMP-9 concentrations in DLBCL and FL. (PDF) [file pone.0078730.s001.pdf]

Clinical significance of sIL-2R levels in B-cell lymphomas

Supplementary information

Methods

Reverse transcriptase–polymerase chain reaction (RT-PCR)

Total cellular RNA was extracted using Trizol (Life Technologies, Gaithersburg, MD) according to the manufacturer's protocol. First-strand cDNA was synthesized from 1 mg of total cellular RNA in a 20-μL reaction volume using an RNA-PCR kit (Takara Shuzo, Kyoto, Japan) with random primers. Thereafter, cDNA was amplified for 30 or 40 cycles for MMP-9 and 28 cycles for β-actin. Oligonucleotide primers were as follows: for MMP-9, sense, 5'- CGCAGACATCGTCATCCAGT-3' and antisense, 5'-GGATTGGCCTTGGAAGATGA-3'; and for β- actin, sense, 5'- ATCTGGCACCACACCTTCTACAATGAGCTG -3' and antisense, 5'- CGTCATACTCCTGCTTGCTGATCCACATCT -3'. Product sizes were 390 bp for MMP-2, 406 bp for MMP-9, and 838 bp for β-actin. Cycling conditions were as follows: denaturing at 95°C for 60 s for MMP-9 and 94°C for 60 s for β-actin, annealing at 62°C for 60 s for MMP-9 and 55°C for 60 s for β-actin, and extension at 72°C for 60 s for MMP-9 and β-actin. PCR products were fractionated on 2% agarose gels and visualized by ethidium bromide staining.

Western blot analysis

Cells were lysed (1% Nonidet P-40, 150mmol/l NaCl, and 0.1% sodium deoxycholate in 20 mmol/l Tris, pH 7.5) in the presence of a protease inhibitor cocktail (Sigma-Aldrich, St. Louis, MI). After 30 min on ice, lysates were centrifuged at 14,000 rpm for 30 min at 4°C and supernatants were collected. Protein (50μg) was separated by 10% or 15% SDS polyacrylamide gel electrophoresis and transferred to a nitrocellulose membrane, immunoblotted with the indicated antibodies, and visualized using enhanced chemiluminescence (ECL) Western blotting detection reagents (Amersham Pharmacia Biotech, Piscataway, NJ). Primary antibodies included anti-MMP-9 (R & D Systems,Inc., Minneapolis, MN) and anti-GAPDH (HyTest Ltd, Turku, Finland).

Cell-conditioned media and gelatin zymography

Human T-cell lines were harvested at the exponential growth phase, washed in serum-free RPMI 1640, and cultured at a concentration of 1×10^6 cells/ml for 72 h. CD19-positive cells of DLBCL, FL and RLH were purified by microbeads and were also cultured in FCS-free RPMI-1640. Cell-conditioned media (supernatants) were collected, concentrated 10-fold using Ultracel YM-30 (Millipore, Billerica, MA, USA)), and were analyzed by zymography according to the manufacturer's instructions (Primary Cell Co., Ltd., Ishikari, Japan)

Immunohistochemistry to detect MMP-9, CD68, and CD163 expression

Samples for histological diagnosis were formalin-fixed, paraffin-embedded and stained using the hematoxylin-eosin (H&E) method. Paraffin sections from each sample were immunostained with mouse monoclonal antibodies against MMP-9 (1:75 dilution; Thermo Fisher Scientific, San Diego, CA), CD163 (Clone 10D6) (1:20 dilution; Thermo Fisher Scientific), and CD68 (KP-1) (1:400 dilution; DakoCytomation, Glostrup, Denmark). CD68 and CD163 antibodies were applied and sections were incubated for 30 min at room temperature. For MMP-9 antibody, sections were treated over-night at 4°C. Biotinylated secondary antibody (10 min for CD68 and CD163, 60 min for MMP-9), followed by peroxidase-labeled streptavidin (10 min for CD68 and CD163, 30 min for MMP-9), was then applied. The period for the diaminobenzidine reaction was 4 min for CD68 and CD163, and less than 1 min for MMP-9.

Analysis of gene expression profiling

For evaluation of gene expression in DLBCL and macrophages/monocytes, previously published data sets were used (GSE11318 [1] and GSE5099 [2]). Data were visualized by Hierarchical Clustering Explorer version 3.0 (http://www.cs.umd.edu/hcil/hce/). To calculate relative gene expression levels, gene expression levels were divided by median expression of each probe.

Supplementary Figure Legends

Figure S1. Expression of CD25 (IL-2Rα) in MCL.
Of note, lymphoma cells in mantle cell lymphoma (MCL) were positive for CD25 (7 of 7 cases; data not shown).

Figure S2. MMP-9 expression in ATL cells.
(A) RT-PCR revealed that 3 of 5 ATL cell lines and ATL patients cell expressed MMP-9.
(B) Cell lysates (100 μg) were blotted with anti-MMP-9 antibody. Patient ATL cells and MT1 expressed MMP-9, while MT2 and HPB-ATL-2 weakly expressed MMP-9.
(C) Zymographic analysis revealed that supernatant after cell culture (1 x 10^6/ml) in FCS-free conditioned medium for 72 h contained pro-MMP-9 in ATL patient cells, MT1 and HPB-ATL-2.

Figure S3. MMP-9 expression in CD19-positive cells from B-cell lymphoma (A) and RLH (B).
Concentrated cell-conditioned media (supernatants) of CD19-positive cells purified from biopsy samples were analyzed by gelatin zymography.

Figure S4. Immunohistochemical staining in intravascular large B cell lymphoma and (IVL) adult T-cell leukemia/lymphoma (ATLL) using anti-MMP-9 antibody.
Lymphoma cells of IVL were negative for MMP-9 (left), while cells of ATLL were strongly positive for MMP-9 (right).

Figure S5. Overall survival based on numbers of CD68 and CD163 positive macrophages in DLBCL total (A), extranodal DLBCL (B) and FL (C).
Patients with DLBCL and FL were divided into two groups based on number of CD68- and CD163-positive macrophages. If the number of CD68- and CD163-positive macrophages was higher or lower than mean number of macrophages, the sample was defined as high or low, respectively.

Figure S6. Gene expression profiles of macrophages and monocytes.
(A) Relative gene expression of CD68, CD163, and proteinases in macrophages/monocytes are shown.
(B) There was a positive correlation between relative expression levels of CD68 and those of MMP-9. These results were evaluated by Pearson correlation coefficient.

(C) Relative expression of CD163 was not proportional to that of MMP-9.

Figure S7. Tumor associated macrophages in diffuse large B-cell lymphoma.

(A) Differences in number of macrophages and sIL-2R levels between GCB-type DLBCL and non-GCB-type DLBCL.

(B) Correlation between number of macrophages and sIL-2R levels in GCB-type DLBCL.

(C) Relative expression profiles of genes related to tumor-associated macrophages (TAMs) [2] and MYC in each type of DLBCL.

ABC; activated B-cell, GCB; germinal center B-cell, PMBL; primary mediastinal B-cell lymphoma.

References

1. Lenz G, Wright GW, Emre NC, Kohlhammer H, Dave SS, et al. (2008) Molecular subtypes of diffuse large B-cell lymphoma arise by distinct genetic pathways. Proc Natl Acad Sci U S A 105: 13520-13525.
2. Solinas G, Schiarea S, Liguori M, Fabbri M, Pesce S, et al. (2010) Tumor-conditioned macrophages secrete migration-stimulating factor: a new marker for M2-polarization, influencing tumor cell motility. J Immunol 185: 642-652.

Mantle cell lymphoma

No. 15

sIL-2R: 1380

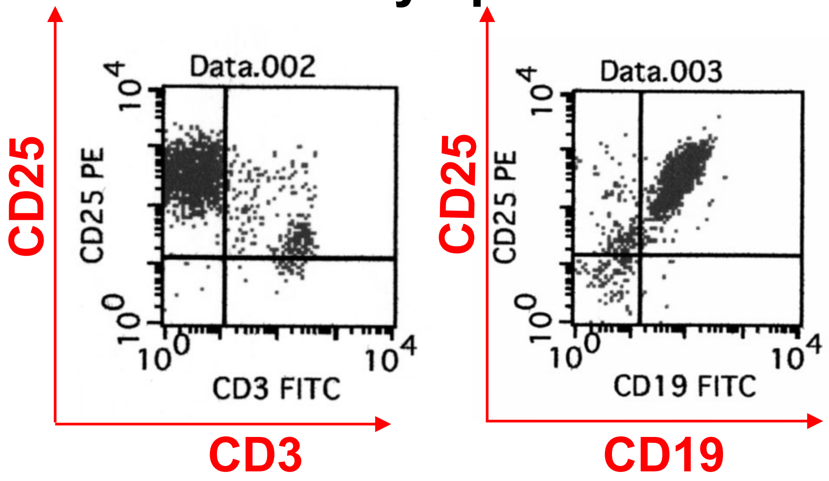

Figure S1

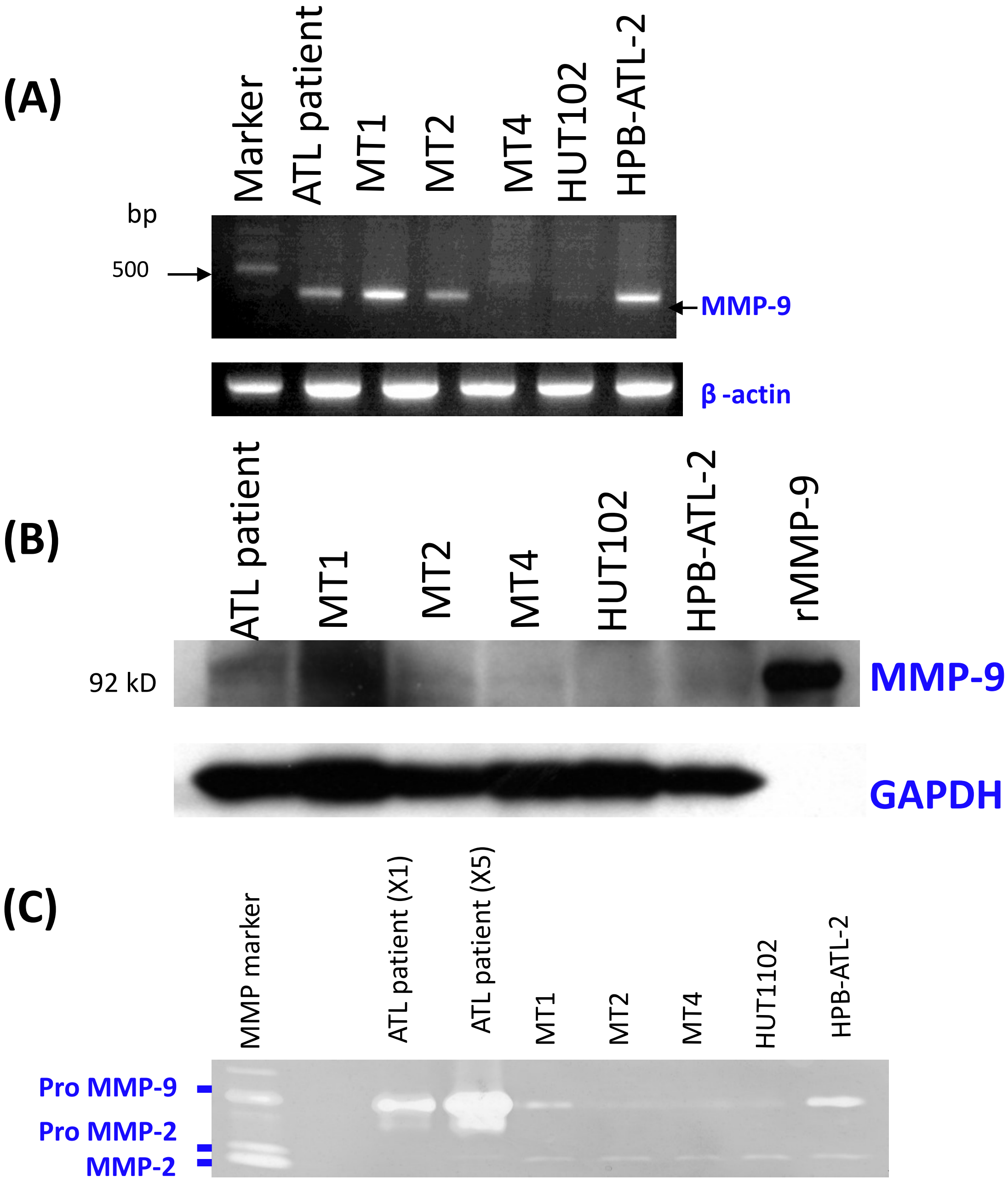
(A)
Marker
ATL patient
MT1
MT2
MT4
HUT102
HPB-ATL-2
bp
500
MMP-9
β -actin
(B)
ATL patient
MT1
MT2
MT4
HUT102
HPB-ATL-2
rMMP-9
92 kD
MMP-9
GAPDH
(C)
MMP marker
ATL patient (X1)
ATL patient (X5)
MT1
MT2
MT4
HUT1102
HPB-ATL-2
Pro MMP-9
Pro MMP-2
MMP-2

Figure S2

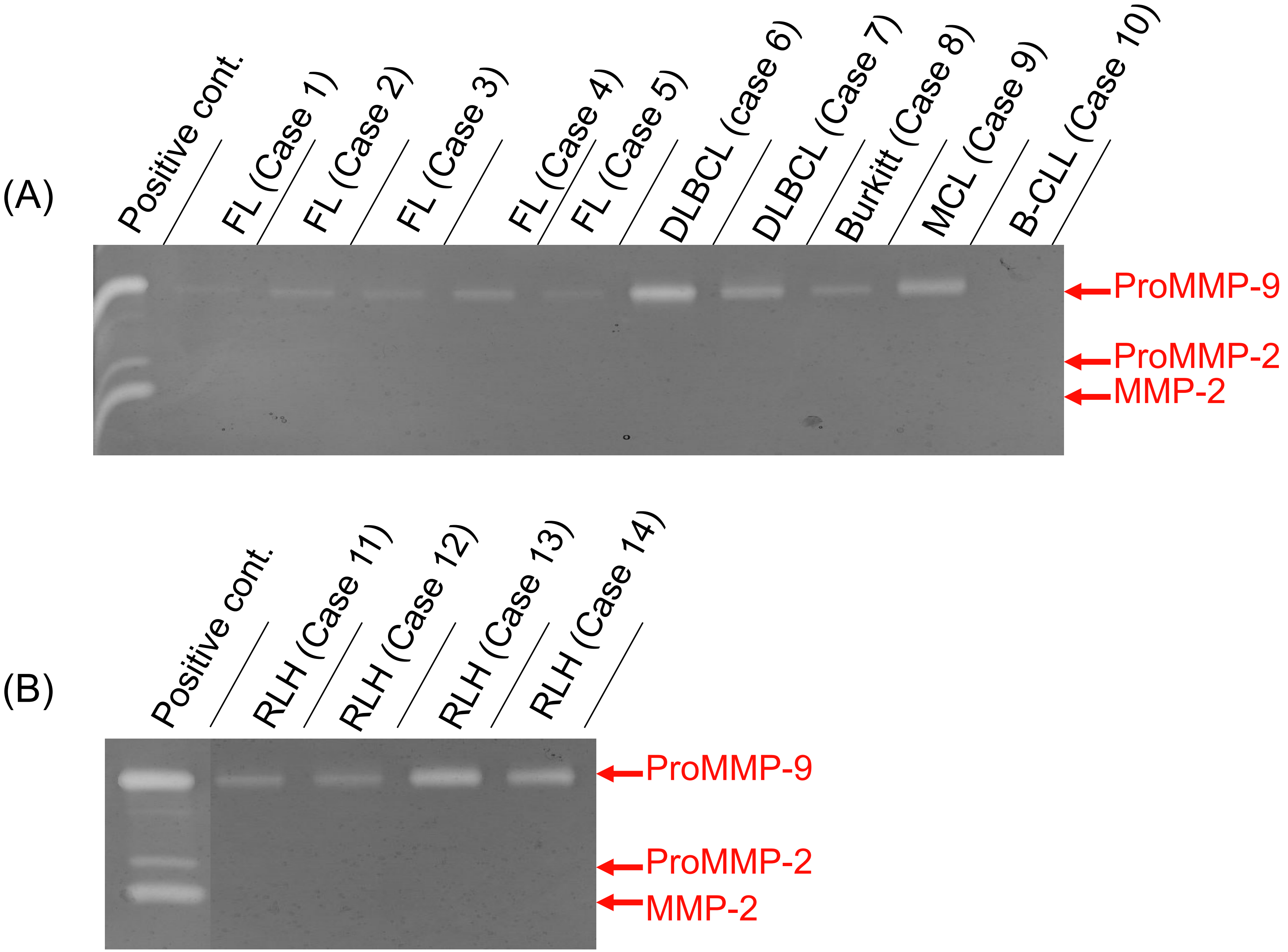

(A)
Positive cont.
FL (Case 1)
FL (Case 2)
FL (Case 3)
FL (Case 4)
FL (Case 5)
DLBCL (case 6)
DLBCL (Case 7)
Burkitt (Case 8)
MCL (Case 9)
B-CLL (Case 10)
ProMMP-9
ProMMP-2
MMP-2
(B)
Positive cont.
RLH (Case 11)
RLH (Case 12)
RLH (Case 13)
RLH (Case 14)
ProMMP-9
ProMMP-2
MMP-2

Figure S3

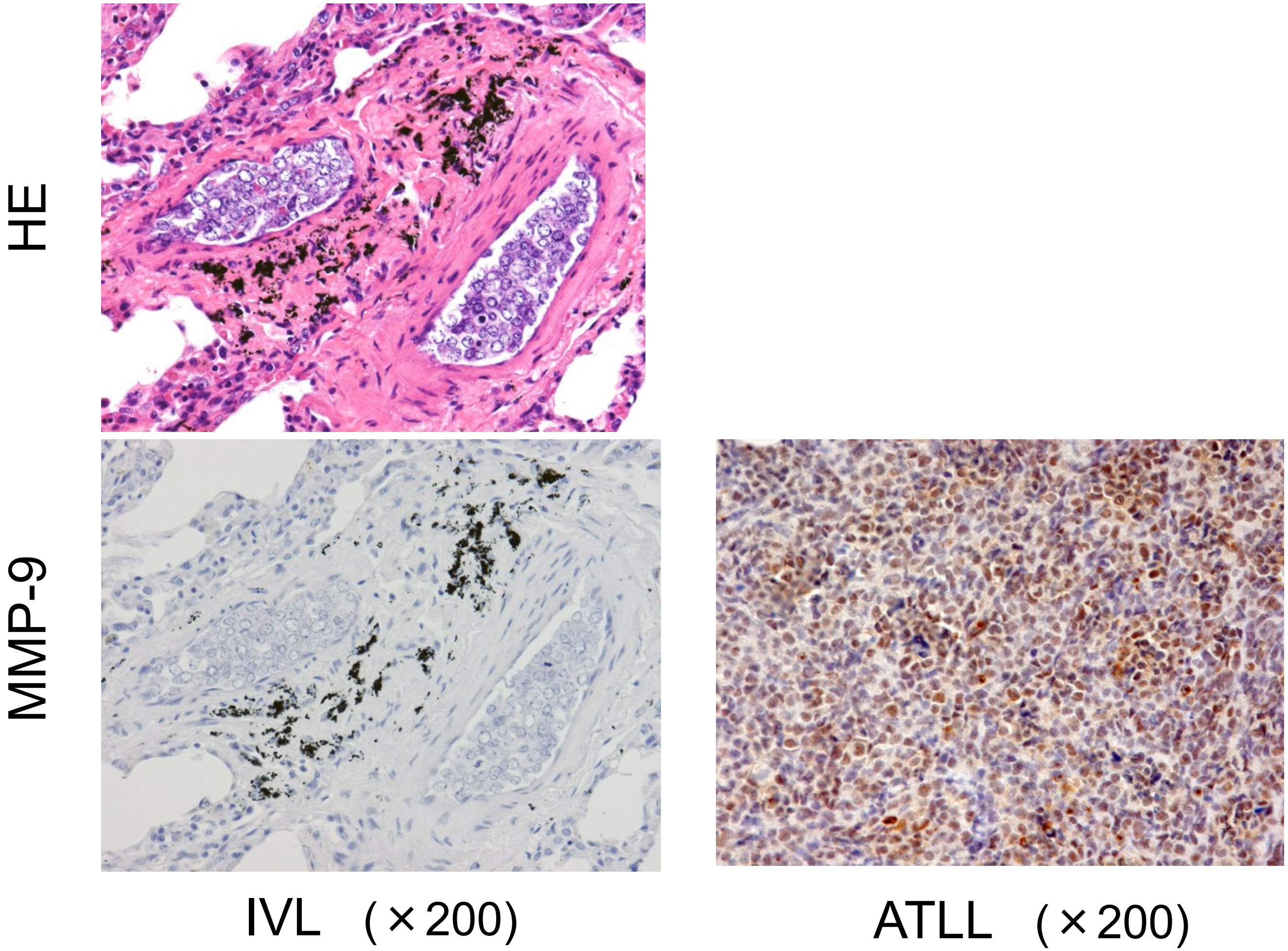

Figure S4

DLBCL n=47

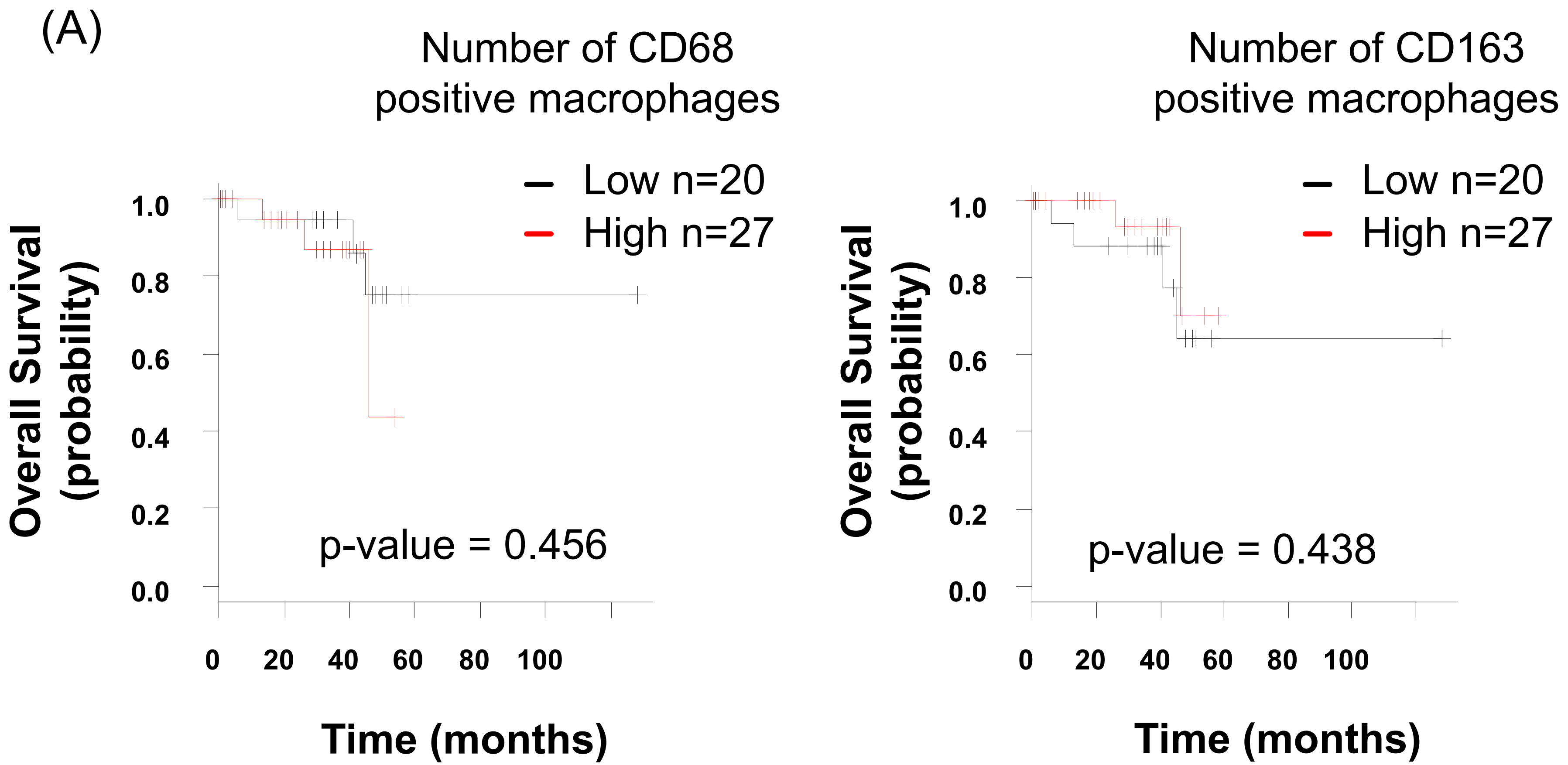

Figure S5

extranodal DLBCL n=19

(B)

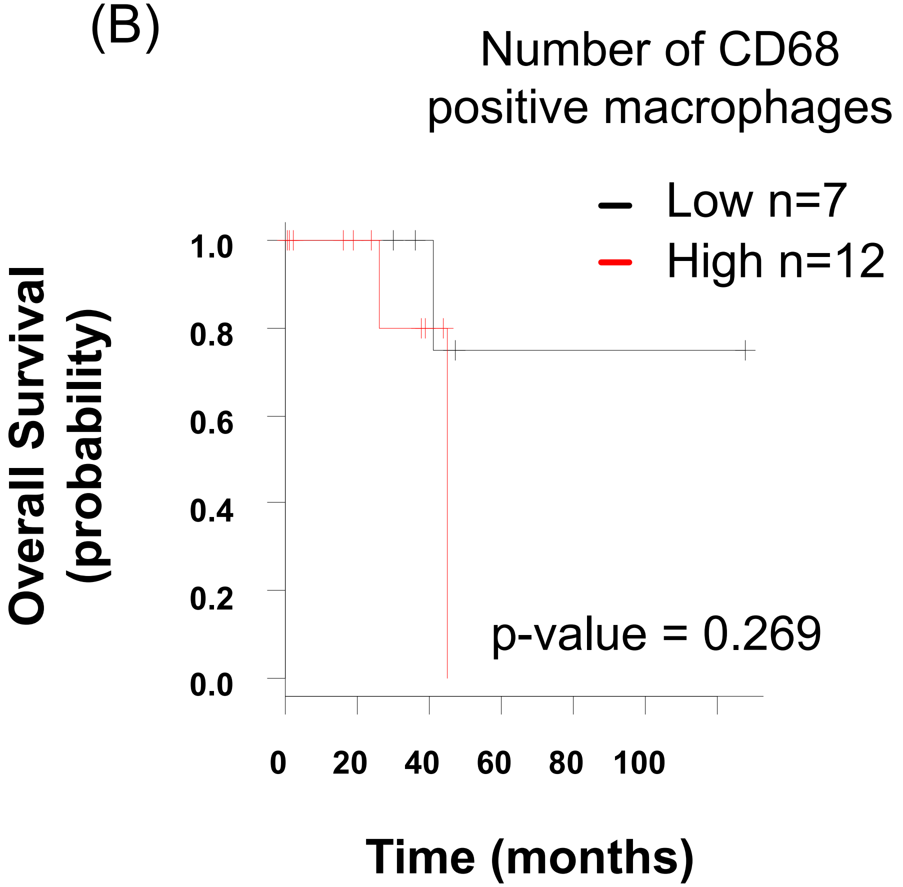

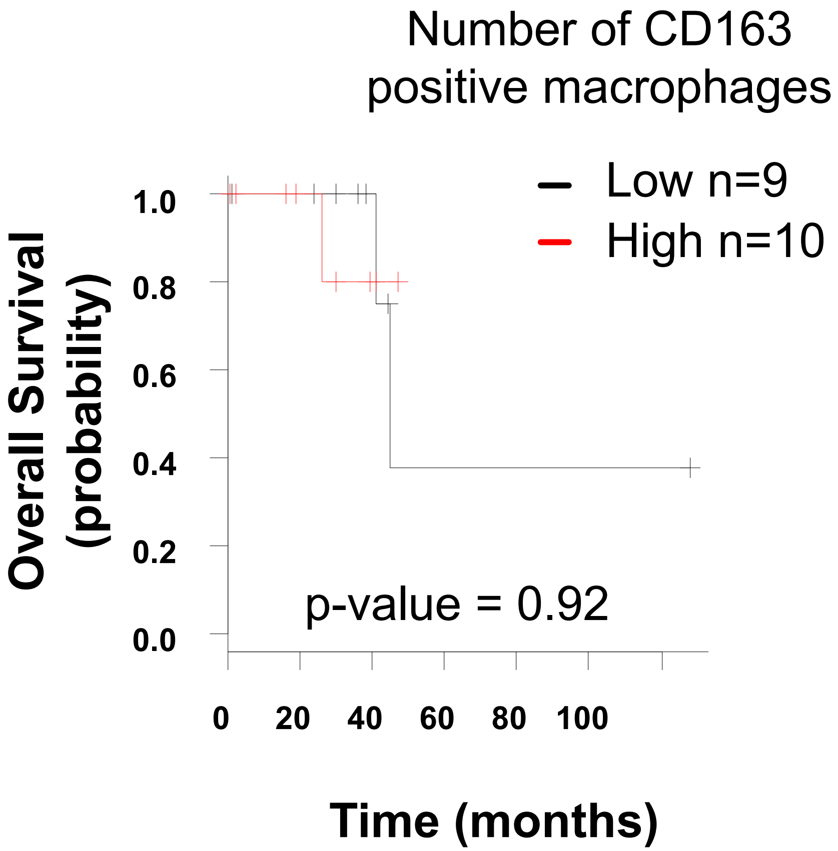

Figure S5

FL n=21

(C)

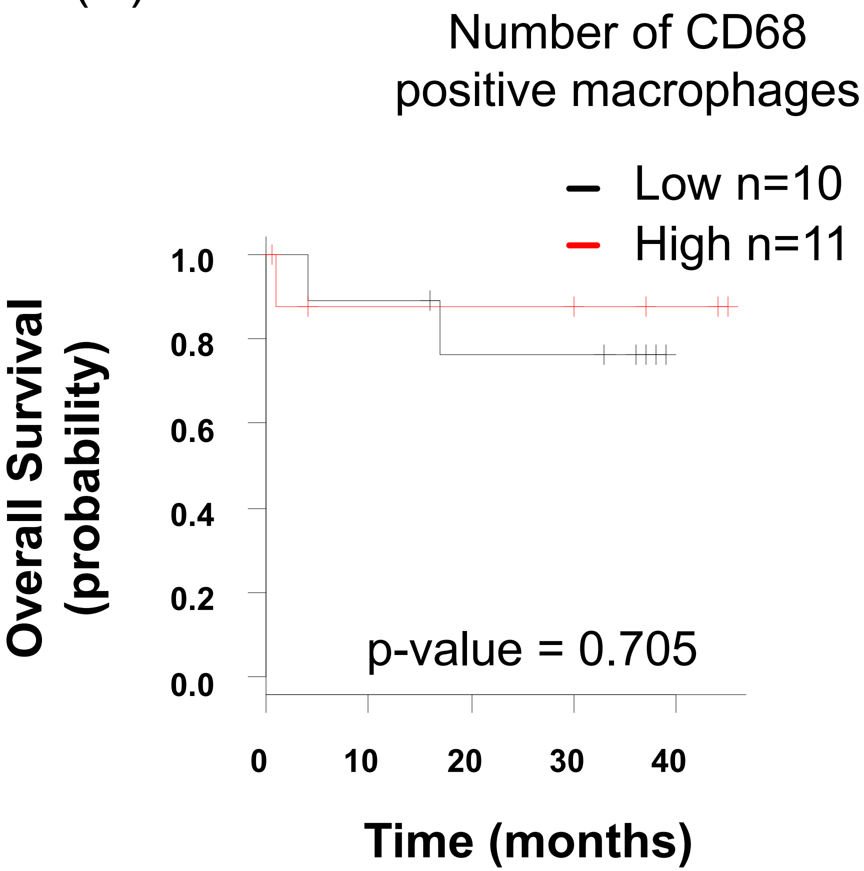

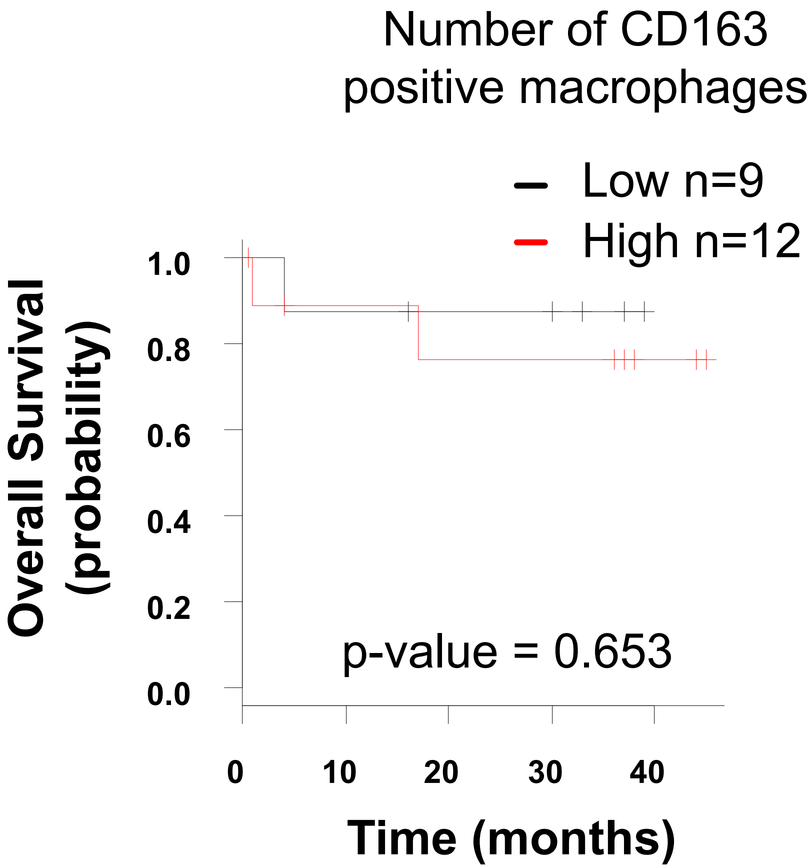

Figure S5

(A)

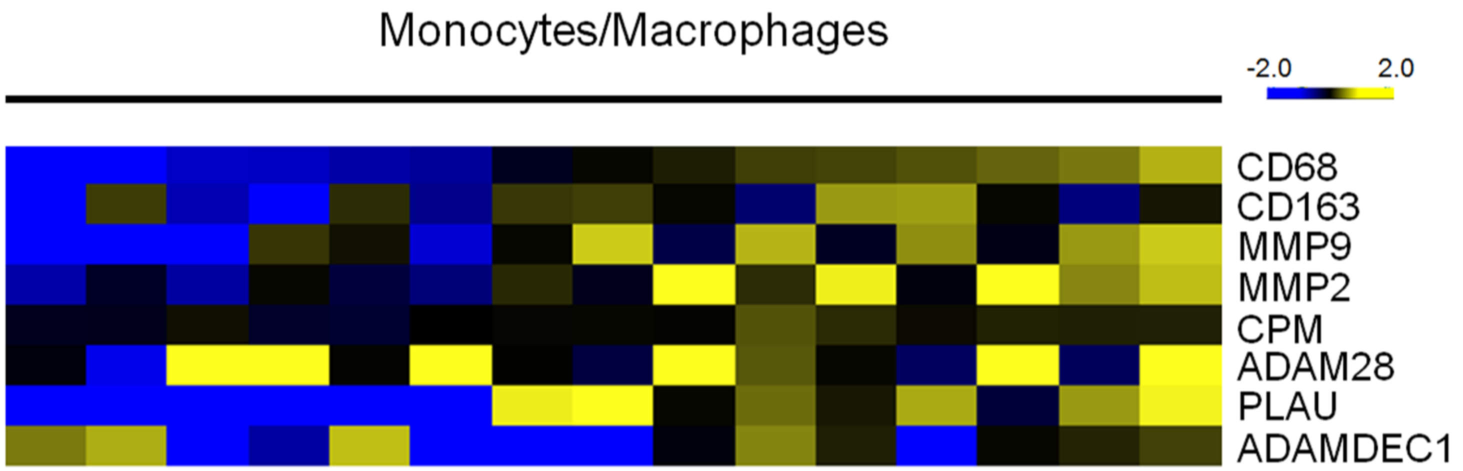

Monocytes/Macrophages
-2.0
2.0
CD68
CD163
MMP9
MMP2
CPM
ADAM28
PLAU
ADAMDEC1

Figure S6

(B)

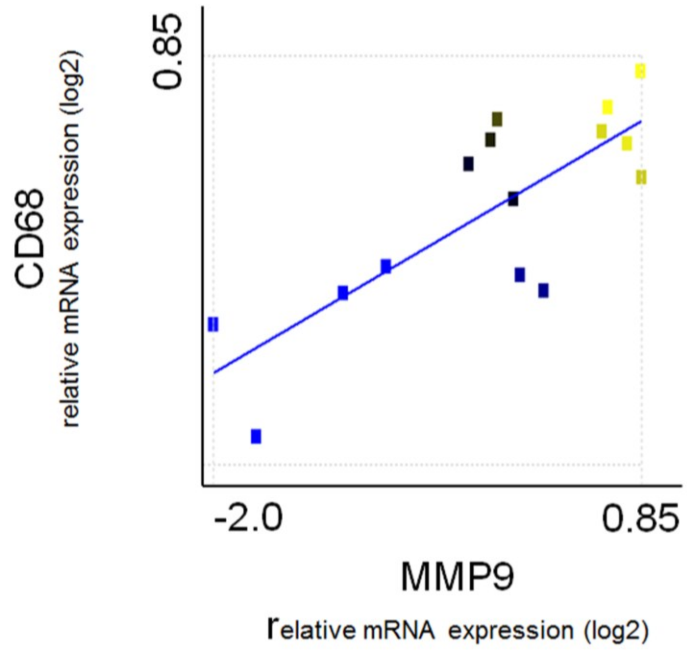

γ^2 = 0.63, p-value = 0.0004

(C)

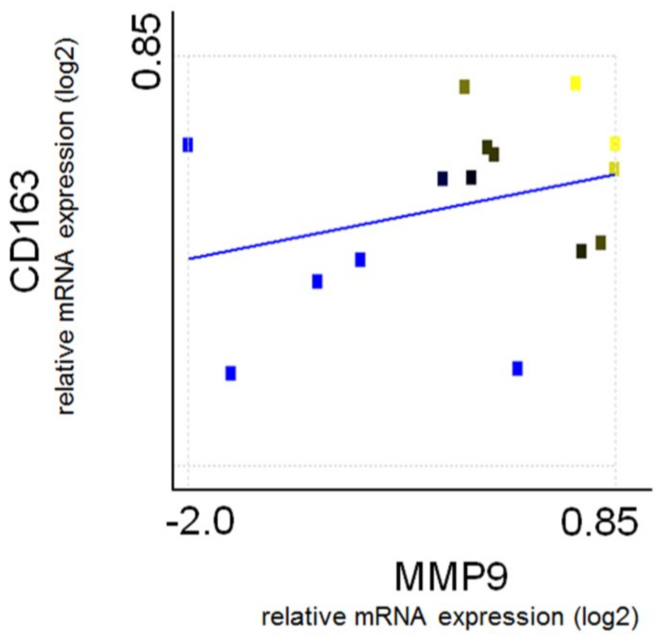

γ^2 = 0.09, p-value = 0.2797

Figure S6

(A)

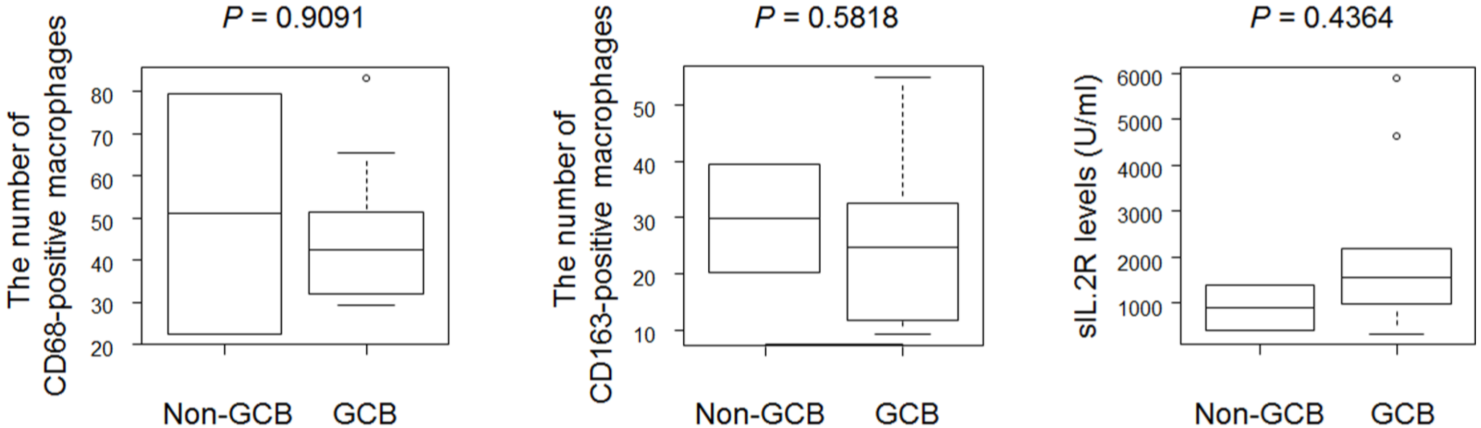

P = 0.9091
The number of CD68-positive macrophages
80
70
60
50
40
30
20
Non-GCB
GCB
P = 0.5818
The number of CD163-positive macrophages
50
40
30
20
10
Non-GCB
GCB
P = 0.4364
sIL.2R levels (U/ml)
6000
5000
4000
3000
2000
1000
Non-GCB
GCB

Figure S7

(B)

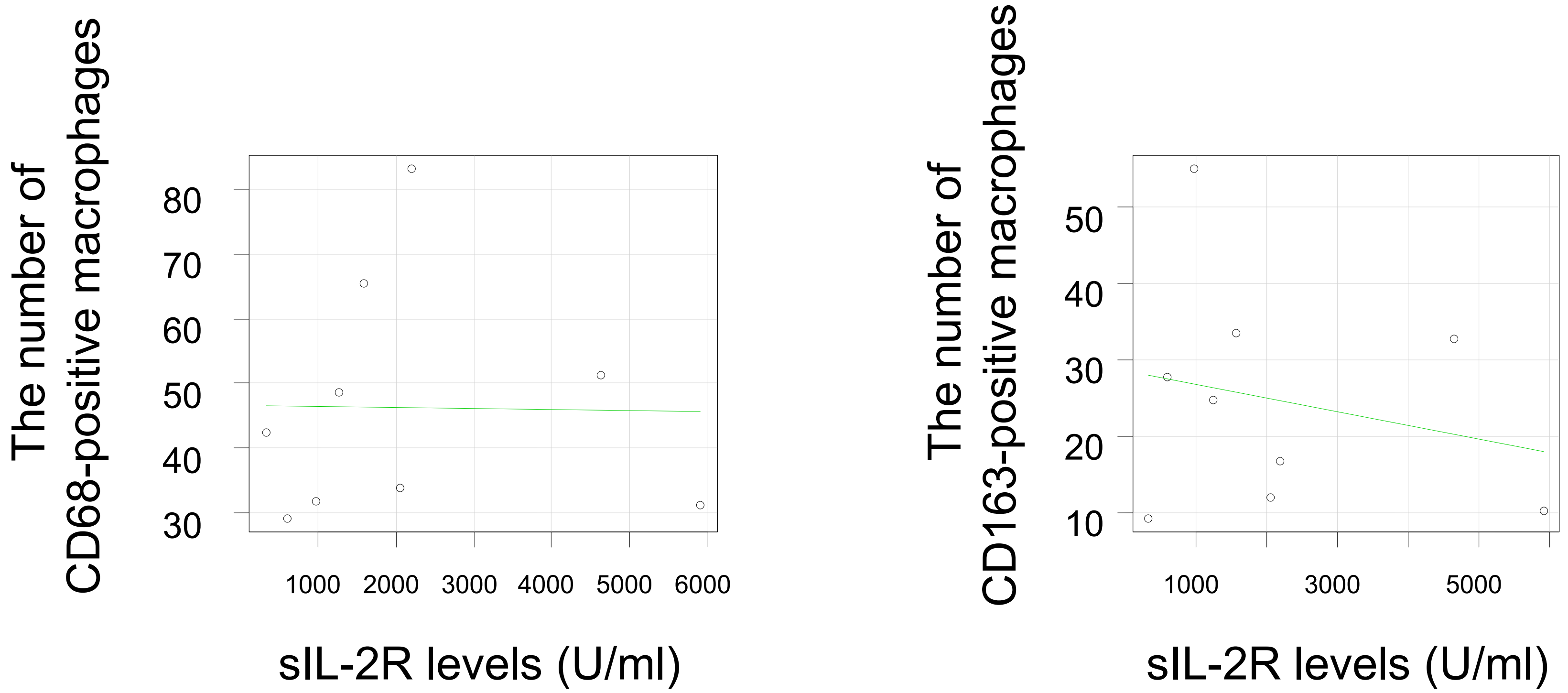

p-value = 0.4933

ρ = 0.2666667

p-value = 0.8432

ρ = -0.08333333

GCB type DLBCL (n=9)

Figure S7

(C)

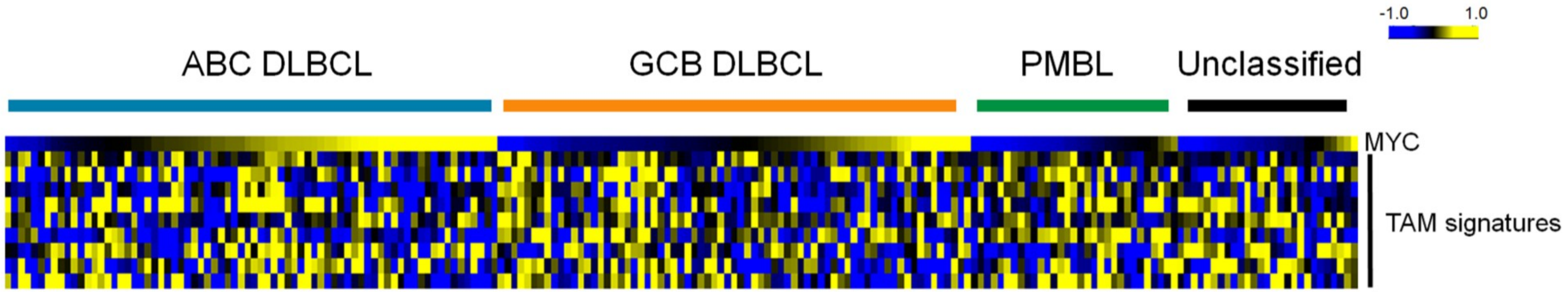
-1.0
1.0
ABC DLBCL
GCB DLBCL
PMBL
Unclassified
MYC
TAM signatures

Figure S7

Table S1 sIL-2R and MMP-9 concentrations in DLBCL and FL

DLBCL				FL		
No	MMP-9 (ng/ml)	sIL-2R (U/ml)		No	MMP-9 (ng/ml)	sIL-2R (U/ml)
1	70.1	8560		1	13.8	768
2	30.1	966		2	128<	6580
3	79.4	680		3	16	1260
4	45.1	907		4	6.4	2902
5	46.1	548		5	32	2419
6	8	763		6	34.9	4333
7	24	860		7	23	751
8	8.32	758		8	45.1	4301
9	57.3	6990		9	128<	4857
10	10.9	534		10	44.2	268
11	8.64	635		11	31	552
12	7.68	571		12	117	1080
13	29.8	5210		13	128<	8273
14	71.7	6812		14	11.5	510
15	22.7	717				
16	32.6	304				
17	37.4	508				
18	83.8	841				
19	36.2	5904				
20	33.6	475				
21	18.2	4960				
22	128	414				
23	128	1928				
24	36.2	2047				
25	12.5	1065				
26	98.6	1134				
27	30.5	5507				
28	82.6	567				
29	44.2	783				
30	94.6	4885				
